# Theranostics for Neuroblastoma: Making Molecular Radiotherapy Work Better

**DOI:** 10.2967/jnumed.124.269121

**Published:** 2025-04

**Authors:** Peter J. Gawne, Helen E. Bryant, Steven G. DuBois, Sally L. George, Juliet Gray, Leona Knox, Kyle B. Matchett, Connie Peet, Katherine A. Vallis, Hugh J. Wallace, Simon Wan, Mark N. Gaze

**Affiliations:** 1 Centre for Cancer Biomarkers and Biotherapeutics, Barts Cancer Institute, Queen Mary, University of London, London, United Kingdom;; 2 UCL Cancer Institute, University College London, London, United Kingdom;; 3 School of Biomedical Engineering and Imaging Sciences, King’s College London, London, United Kingdom;; 4 School of Medicine and Population Health, University of Sheffield, Sheffield, United Kingdom;; 5 Dana-Farber/Boston Children’s Cancer and Blood Disorders Center, Harvard Medical School, Boston, Massachusetts;; 6 Division of Clinical Studies, Institute of Cancer Research, London, United Kingdom;; 7 Children and Young People’s Unit, Royal Marsden Hospital, London, United Kingdom;; 8 Centre for Cancer Immunology, University of Southampton, Southampton, United Kingdom;; 9 Solving Kids’ Cancer, London, United Kingdom;; 10 Personalised Medicine Centre and Clinical Translational Research and Innovation Centre, Altnagelvin Area Hospital, School of Medicine, Ulster University, Derry-Londonderry, United Kingdom;; 11 Department of Radiotherapy, University College London Hospitals NHS Foundation Trust, London, United Kingdom;; 12 Department of Oncology, University of Oxford, Oxford, United Kingdom;; 13 Paediatric Nuclear Medicine, Royal Hospital for Children, Glasgow, United Kingdom;; 14 Department of Clinical Physics and Bioengineering, NHS Greater Glasgow and Clyde, Glasgow, United Kingdom;; 15 University of Glasgow, Glasgow, United Kingdom;; 16 Institute of Nuclear Medicine, University College London Hospitals NHS Foundation Trust/University College London, London, United Kingdom; and; 17 Department of Oncology, University College London Hospitals NHS Foundation Trust, London, United Kingdom

**Keywords:** molecular radiotherapy, neuroblastoma, radionuclide therapy, radiopharmaceutical therapy, theranostics

## Abstract

Despite improvements in neuroblastoma treatment, survival figures lag behind those of many other childhood malignancies. New treatments, and better use of existing treatments, are essential to reduce mortality. Neuroblastoma expresses several molecular targets for radionuclide imaging and therapy, of which the most widely exploited is the norepinephrine transporter. [^123^I]metaiodobenzylguanidine (MIBG) imaging and [^131^I]MIBG treatment, which target this physiologic pathway, have been in clinical practice for 40 y. Although therapy outcomes have been favorable, [^131^I]MIBG use has not yet been optimized. Somatostatin receptors and the disialoganglioside are alternative targets, but their use remains experimental. The charity Children’s Cancer Research Fund organized a workshop bringing together a broad range of scientists including radiochemists, radiobiologists, radiation physicists, clinical researchers including pediatric oncologists and nuclear medicine physicians, and patient advocates from the United Kingdom, United States, and continental Europe to share their experiences with molecular imaging and radiotherapy of neuroblastoma and discuss potential ways of improving treatment outcomes and access. These include development of alternative vectors targeting somatostatin receptors and disialoganglioside, isotopes such as α-particle and Auger electron emitters with different radiation characteristics, and combinations with external-beam radiotherapy, immunotherapy, and DNA damage repair inhibitors. Barriers to progress discussed included the unpredictable radioisotope supply, production of novel radiopharmaceuticals, lack of data regarding which are the best combination therapies, and insufficient clinical facilities. The aim was to stimulate the development and assessment of more effective treatments.

Approximately half the children with neuroblastoma have high-risk disease (1). Despite therapeutic advances, survival is poor (2). Neuroblastoma is characterized by biochemical pathways and cell membrane molecules not found in most normal tissues (3), providing opportunities for nuclear medicine imaging and molecular radiotherapy (MRT).

[^123^I]metaiodobenzylguanidine (MIBG) is the gold standard for imaging neuroblastoma, and semiquantitative scoring systems are of prognostic value (4). Labeled with a β-emitting isotope, [^131^I]MIBG MRT is used to treat neuroblastoma (5). Despite 40 y of clinical experience, [^131^I]MIBG therapy is not yet regarded as a standard first-line treatment. Various strategies have been explored to try and improve outcomes. Use of vectors aimed at other molecular targets, or radionuclides emitting radiation with different characteristics, may also be advantageous (6). Progress in imaging technology combined with innovative radiotracers may allow for improved disease assessment (7,8).

## MATERIALS AND METHODS

The charity Children’s Cancer Research Fund (https://www.childrenscancerresearchfund.co.uk/) convened a symposium in Manchester, U.K., in September 2024. Twenty-one invited speakers included pediatric oncologists (5); cellular, radiation, and molecular biologists (4); nuclear medicine physicians (3); radiochemists (3); radiation (clinical) oncologists (2); a pediatric surgeon; a nuclear medicine physicist; a therapeutic radiographer; and a parent of a child with neuroblastoma who had received MRT for neuroblastoma (speaker list in supplemental materials, available at http://jnm.snmjournals.org). In addition, there was an invited audience of 20 people including clinicians, scientists, and parents mirroring the mix of speakers. In addition to questions and debate after each presentation, informal networking continued in the breaks, with the aim of creating new research proposals and collaborations.

## RESULTS

### Imaging and Therapy Targeting the Norepinephrine Transporter

The physiologic pathway that has been most exploited is the norepinephrine transporter, expressed in over 90% of neuroblastoma patients, which can be targeted using MIBG.

[^123^I]MIBG planar scintigraphy and SPECT/CT is the standard imaging technique for neuroblastoma, including selection of patients for [^131^I]MIBG therapy (9). This 2-d procedure is a 45-min scan requiring general anesthesia in younger patients performed 24 h after radiopharmaceutical injection. Thyroid blockade is coadministered to reduce uptake of free iodide by the thyroid gland (10). PET/CT with [^18^F]FDG is recommended when the tumor is [^123^I]MIBG-negative (11). However, it is a metabolic tracer not conducive to theranostics.

PET/CT with [^18^F]metafluorobenzylguanidine is a single-day procedure with scanning an hour after injection; resolution is much greater, identifying more lesions, and thyroid blockade is not required (12). The short half-life of ^18^F (110 min) may require national production of the tracer [^18^F]metafluorobenzylguanidine because of the potential time taken for international supply lines.

The positron emitter ^124^I is an alternative to ^123^I, and [^124^I]MIBG can be visualized on PET/CT. The longer half-life (4.2 d) allows sequential scanning over days, and measurement of retention in tissues over time enables prediction of tumor and normal-organ radiation doses that would follow [^131^I]MIBG therapy (13).

The introduction of long-axis-field-of-view (total-body) PET/CT technology with exquisite sensitivity and spatiotemporal resolution expands the technologic constraints on the 3-way trade-off between image quality, scan duration, and radiation dose reduction. This may reduce scan time so that general anesthesia may be unnecessary (14).

There have been several clinical trials of [^131^I]MIBG therapy over 4 decades; mostly early-phase studies on refractory or relapsed high-risk neuroblastoma. The main exception is the Children’s Oncology Group phase III randomized trial ANBL1531 (NCT03126916), a comparison of the addition of [^131^I]MIBG therapy to standard induction therapy. Recruitment is complete; results are not yet available. Recent clinical trials of MRT are detailed in Table 1.

**TABLE 1.** Recent MRT Trials for Neuroblastoma

Trial	Title and location	Type and date	Status
Completed and published			
LuDO	Phase IIa trial of MRT with ^177^Lu-DOTATATE in children with primary refractory or relapsed high-risk neuroblastoma (University of Birmingham)	[^177^Lu]DOTATATE, phase II, 2013–2017	Published 2020 (*27*)
NANT 2011-01 (NCT02035137)	Randomized phase II pick winner study of ^131^I-MIBG, ^131^I-MIBG with vincristine and irinotecan, or ^131^I-MIBG with vorinostat for resistant/relapsed neuroblastoma (NANT)	[^131^I]MIBG, randomized phase II, 2014–2019	Published 2021 (*37*)
MIITOP (NCT00960739)	Phase II study of ^131^I-metaiodobenzylguanidine with 5 d of topotecan for refractory or relapsed neuroblastoma (Centre Oscar Lambret)	[^131^I]MIBG, phase II, 2008–2015	Published 2023 (*21*)
Accrual completed—awaiting maturation of data			
ANBL1531 NCT03126916	Testing addition of ^131^I-MIBG or lorlatinib to intensive therapy in people with high-risk neuroblastoma (Children’s Oncology Group)	[^131^I]MIBG, randomized phase III, 2018–2024	Closed to accrual
MINIVAN (NCT02914405)	Phase I study of ^131^I MIBG followed by nivolumab and dinutuximab β-antibodies in children with relapsed/refractory neuroblastoma (University Hospital Southampton)	[^131^I]MIBG, phase I, 2018–2024	Closed to accrual
OPTIMUM (NCT03561259)	Phase II, open label, 2-arm study of therapeutic iobenguane (^131^I) as single agent or in combination with vorinostat for recurrent or progressive high-risk neuroblastoma subjects (Jubilant DraxImage Inc.)	[^131^I]MIBG, nonrandomized phase II, 2019–2023	Closed to accrual
VERITAS (NCT03165292)	Evaluation of 2 intensification treatment strategies for neuroblastoma patients with poor response to induction (European Neuroblastoma Clinical Trials Group)	[^131^I]MIBG, randomized phase II, 2018–2023	Closed to accrual (terminated prematurely)
Omburtamab radioimmunotherapy (NCT03275402)	^131^I-omburtamab radioimmunotherapy for neuroblastoma central nervous system/leptomeningeal metastases (Y-mAbs Therapeutics)	^[131^I]-omburtamab, phase II, 2018–2023	Closed to accrual
NANT 2017-01 (NCT03332667)	MIBG with dinutuximab ± vorinostat (NANT)	[^131^I]MIBG, phase I, 2018–2023	Closed to accrual
Open and recruiting			
LuDO-N (NCT04903899)	^177^Lu-DOTATATE in children with primary refractory or relapsed high-risk neuroblastoma (Karolinska institute)	[^177^Lu]DOTATATE, phase II	Open since 2021 and recruiting
NEUROBLU 02 (NCT03966651)	A clinical study evaluating safety of peptide receptor radionuclide therapy (PRRT) with ^177^Lu-DOTA^0^-Tyr^3^-octreotate in children with refractory or recurrent neuroblastoma expressing somatostatin receptors (Institut Claudius Regaud)	[^177^Lu]DOTATATE, phase I	Open since 2023 and recruiting
GD2-SADA: ^177^Lu-DOTA complex (NCT05130255)	GD2-SADA: ^177^Lu-DOTA complex in patients with solid tumors known to express GD2 (Y-mAbs Therapeutics)	[^177^Lu] 2-step radioimmunotherapy, phase I	Open since 2022 and recruiting; amended 2024 to include neuroblastoma patients ≥ 18 y
Planned			
MINT	Biomarker-enriched phase I/II clinical trial of ^131^I-MIBG therapy with talazoparib for treatment of relapsed or refractory neuroblastoma (University of Birmingham)	[^131^I]MIBG, phase I/II	Funded, pending regulatory approval

A review of [^131^I]MIBG therapy reported response rates varying from 0% to 75% (mean, 32%). This wide range was due to a highly variable case mix, highly variable administration schedules, and no use of standardized response criteria (5). Another review concluded that [^131^I]MIBG therapy can be an effective treatment to reduce tumor burden in about one third of patients (15).

Administration of [^131^I]MIBG therapy to children faces various logistic challenges. These include irregularities in radiopharmaceutical supply, the need for specialized inpatient facilities in a pediatric environment, provision for accommodation and training in radiation protection for adult carers, regular staff training, a prolonged admission for radiation protection, radioactive waste storage and disposal, and the requirement for multiple-time-point scanning, often under general anesthesia, for dosimetry. In addition, medical complications require more complicated management when the patient is highly radioactive. Nausea and vomiting are usually prevented with prophylactic antiemetics. Myelosuppression is common, requiring regular blood tests while the child is radioactive, necessitating special handling in the laboratory, and may indicate the need for blood and platelet transfusions. For whole-body radiation doses exceeding 2 Gy, hematopoietic stem cell transfusion is usually required.

### Administered Activity, Whole-Body Dosimetry, and Tumor Dosimetry

There are different administration schedules in use for MRT. For instance, a fixed administered activity may be used, regardless of the size of the patient. In some studies, the administered activity is adjusted by weight.

However, the amount of radioactivity administered, even if weight-adjusted, does not result in a uniform radiation dose to the whole body (16). This lack of uniformity is because of differing kinetics between patients, due to a heterogeneous burden of disease, and varying avidity of and retention by neuroblastoma cells for the radiopharmaceutical, which may change even within the same course of treatment (17). It may be helpful to standardize whole-body dose, as this is a proxy for toxicity (18). Standardization can be achieved by initially administering a weight-based activity, measuring the resulting whole-body radiation dose received, and administering a second activity calculated to raise the total whole-body dose from the 2 administrations combined to a desired level (19). This method has been used in several clinical trials, including MINIVAN (NCT02914405), with a prescribed whole-body dose of 2 Gy to avoid the need for stem cell support, and MIITOP (NCT00960739) and VERITAS (NCT03165292), with a prescribed whole-body dose of 4 Gy (20). It is not known if this split administration strategy achieves more favorable outcomes. Even when the whole-body dose is standardized, the tumor dose may vary by an order of magnitude (21). This variation matters, as response relates to tumor dose received (22). Accurate tumor dosimetry is therefore highly desirable, despite the additional practical difficulties inherent in younger children such as the need for serial imaging under general anesthesia.

**NOTEWORTHY**

- Several theranostic pairs are available to image and treat neuroblastoma.
- Various clinical trials are under way to evaluate novel MRT agents and combinations.
- Further research is required to optimize treatments.

### Alternative Targets for Molecular Imaging and Radiotherapy in Neuroblastoma

**FIGURE 1.**
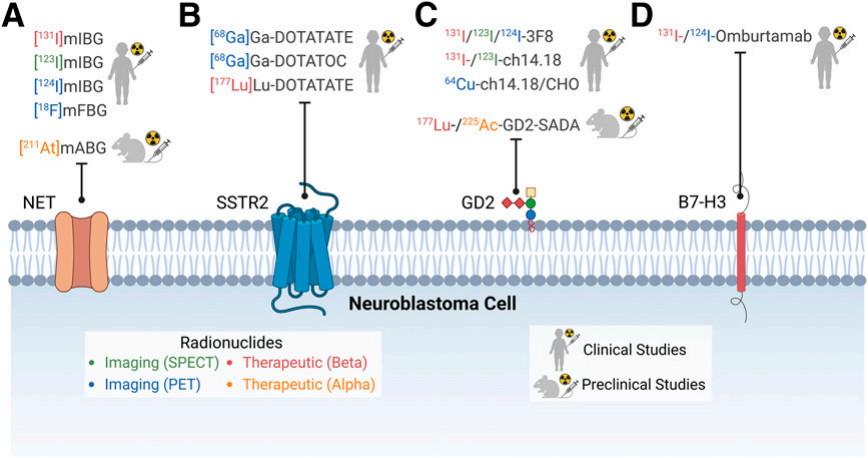
Imaging and therapeutic radiopharmaceuticals for treating neuroblastoma and their molecular targets. (A) Norepinephrine transporter targeted by radioiodinated and radiofluorinated metabenzylguanidine analogs. (B) Somatostatin receptor 2 can be targeted by radiolabeled peptides DOTATOC and DOTATATE for PET imaging and therapy. (C) GD2 has been targeted using variety of radiolabeled antibodies, 3F8, dinutuximab (ch14.18), and dinutuximab β (ch14.18/CHO), as well as using self-assembling and disassembling bispecific antibody for 2-step pretargeted radioimmunotherapy. (D) Finally, B7-H3 has been targeted with radioiodinated antibody, omburtamab. B7-H3 = B7 homolog 3; GD2 = disialoganglioside; NET = norepinephrine transporter; SADA = self-assembling and disassembling; SSTR2 = somatostatin receptor 2. (Created in BioRender; https://BioRender.com/j40j096.)

Alternative molecular targets in neuroblastoma can be imaged (Fig. 1). The somatostatin receptor, particularly subtype 2, is frequently expressed (23). [^68^Ga]Ga-DOTATATE or DOTATOC PET CT is used to show and quantify the distribution of somatostatin receptors on neuroblastoma (24). Interestingly, sometimes disparate distributions from [^123^I]MIBG scans are apparent, indicating the phenotypic heterogeneity of different neuroblastoma deposits (25).

Demonstration of somatostatin receptor avidity on [^68^Ga]Ga-DOTATATE PET scans allows treatment with the radiolabeled somatostatin analog [^177^Lu]Lu-DOTATATE (26). An initial clinical trial showed limited antitumor activity, though several newer trials evaluating [^177^Lu]Lu-DOTATATE for neuroblastoma are in progress: LuDO-N (NCT04903899) and NEUROBLU-2 (NCT03966651). At present, [^177^Lu]Lu-DOTATATE MRT for neuroblastoma is experimental and should be used only in clinical trials.

Neuroblastoma cells also highly express disialoganglioside GD2, which is a well-established immunotherapy target with monoclonal antibodies including dinutuximab, dinutuximab β, and naxitamab (hu3F8) (27). Anti-GD2 antibodies have been radiolabeled for theragnostic applications. Radioimmunotherapy using [^131^I]3F8 was initially explored in neuroblastoma patients but with dose-limiting myelotoxicity (28). More recently, [^131^I]dinutuximab was evaluated (29). In addition to MRT, clinical PET/MRI of GD2 expression of neuroblastoma lesions using [^64^Cu]Cu-dinutuximab β has recently been reported (30*,*^31^) and may allow stratification of anti-GD2 therapies based on tumor lesion GD2 expression in patients.

Finally, neuroblastomas also commonly express B7 homolog 3, which is a cell surface immunoregulatory glycoprotein. A radiolabeled antibody 8H9 ([^131^I]omburtamab) has been evaluated, mainly in children with central nervous system relapsed neuroblastoma (NCT03275402) (32).

### Radiation Sensitizers

The coadministration of various drugs alongside MRT has been shown in preclinical studies and sometimes clinical studies to enhance its cytotoxicity. This occurs via increasing the amount of DNA damage, inhibiting repair of DNA damage, or redistributing cells into radiation-sensitive phases of the cell cycle. Examples include the camptothecin-derived topoisomerase I inhibitors topotecan and irinotecan (33), which both increase DNA damage and inhibit repair (34). There is preclinical evidence of synergy with [^131^I]MIBG therapy (35). Combinations of [^131^I]MIBG therapy with topotecan or irinotecan have been explored in clinical trials (20*,*^36^). Preclinical research has shown that the histone deacetylase inhibitor vorinostat increases expression of functional norepinephrine transporter and decreases expression of DNA damage repair proteins in neuroblastoma (37*,*^38^). The MIITOP study of [^131^I]MIBG therapy with topotecan reported an objective response rate of 13%, but there was no comparator arm (20). The NANT 2011-01 trial reported objective response rates of 14% for both the [^131^I]MIBG-alone arm and the [^131^I]MIBG-with-vincristine-and-irinotecan arm and 32% for the [^131^I]MIBG-with-vorinostat arm (36).

Poly(adenosine diphosphate ribose)polymerase inhibitors, such as olaparib and talazoparib, also potentiate cytotoxicity in experimental models (39*,*^40^). They may have increased benefit in neuroblastoma patients with homologous recombination repair pathway alterations, such as *ATRX* mutations (41) or germline *BARD1* variants (42). Others have shown that increased levels of oncogene-induced replication stress (e.g., *MYCN* amplification (43)) also result in preclinical sensitivity to poly(adenosine diphosphate ribose)polymerase inhibitors. The use of olaparib with [^131^I]MIBG therapy and subsequent maintenance talazoparib has been reported (44). A clinical trial of [^131^I]MIBG therapy with talazoparib is in preparation.

For optimal outcomes, DNA repair inhibitors are desirable, as they sensitize—to radiation damage—tumor cells differently from normal tissue. Otherwise, the effect is simply equivalent to dose escalation with no change in the therapeutic index. In addition to poly(adenosine diphosphate ribose)polymerase inhibitors, polymerase θ inhibitors are in this category and offer a novel prospect of synergy with MRT but have yet to be investigated for neuroblastoma (45*,*^46^).

### Combinations with Immunotherapy

There is preclinical evidence of a complex interplay between radiation effects and response to immunotherapy (47). Empiric support for the concept that MRT potentiates immunotherapy can be found in the significantly superior outcomes of patients with relapsed neuroblastoma who received [^131^I]MIBG therapy before allogeneic bone marrow transplantation and dinutuximab β-immunotherapy, compared with those treated with allogeneic transplantation and immunotherapy only (48). One current trial, MINIVAN (NCT02914405), is evaluating treatment of patients with [^131^I]MIBG therapy before double immunotherapy with dinutuximab β and the anti–programmed cell death protein 1 monoclonal antibody nivolumab. Another trial (NCT03332667) evaluated [^131^I]MIBG with dinutuximab with and without vorinostat. Results of both trials are awaited.

### Combining External-Beam and Molecular Radiotherapy

External-beam radiotherapy to the primary tumor site is part of standard treatment for high-risk neuroblastoma. However, as most patients have disseminated disease, there is a rationale for combining external-beam radiotherapy with MRT, which simultaneously targets metastatic deposits. An important benefit of this combination is that external-beam radiotherapy and MRT have nonoverlapping toxicity profiles. This means that it may not be necessary to compromise on the administered dose of either component. Sequencing of the 2 treatments is likely to be important. For example, preclinical research suggests that external irradiation of a tumor alters blood vessel permeability, which in turn may enhance tumor uptake of subsequently delivered MRT (49). Conversely, however, external radiation may provoke intratumoral inflammation, fibrosis, or an increase in the hypoxic fraction among surviving cells, leading to impaired tumor uptake or reduced radiotoxicity of subsequently administered MRT. Although there is currently a dearth of research into the optimization of external-beam radiotherapy and MRT combinations in neuroblastoma, preclinical research in other tumor types has shown that maximum benefit is usually achieved when the 2 treatments are given synchronously rather than sequentially (50).

A computational system that allows absorbed radiation dose from both sources to be summed, and that considers the different radiobiologic effects of both treatments, is needed to safely progress this therapeutic strategy (51).

### Alternative Radionuclides

Most MRT to date has involved β-emitting radionuclides such as ^131^I and ^177^Lu. Their physical and radiobiologic properties are well understood, and they are clearly effective. However alternative radionuclides that emit α-particles or Auger electrons may have advantages in certain situations.

α-particles, emitted by radionuclides including ^223^Ra, ^211^At, ^212^Pb, and ^225^Ac, offer at least 2 potential advantages over β-emitters. First, they cause many more ionization events along their pathlength. This higher-linear-energy transfer causes greater DNA damage with more double-strand breaks and a lower chance of repair. Consequently, cells are more likely to be killed. Second, because their pathlength is much shorter than that of β-particles (simply a few cell diameters), a greater proportion of the energy released is located within smaller micrometastases than is the case with, for example, ^131^I. Although an analog of MIBG labeled with ^211^At, metaastatobenzylguanidine, was first considered for use in neuroblastoma over 30 y ago but not developed further, there has recently been a resurgence of interest in its possible benefits (6). The shorter pathlength of these α-particles may reduce hospital time and the need for lead shielding compared with β-emitters.

Compared with α-emitters, Auger electrons emitted by radionuclides such as ^125^I, ^201^Tl, and ^111^In have even shorter pathlengths but also have high-linear-energy transfer. If localized in the nucleus of a cell—particularly if incorporated into DNA—or on the cell membrane, they are highly toxic. Additionally, Auger electrons may start a strong bystander response leading to cell death (52). Although an interesting field of research, the use of Auger-emitting radiopharmaceuticals has yet to find a place in the MRT of neuroblastoma.

### Radionuclide Supply and Radiopharmaceutical Availability

One of the biggest challenges facing the development of, and treatment with, MRT for neuroblastoma is the availability of radiopharmaceuticals. For example, [^18^F]metafluorobenzylguanidine and [^124^I]MIBG, mentioned above for imaging use, are not commercially available. There are worldwide shortages of nuclear reactors producing radioisotopes, and many existing reactors are nearing the end of their lives, without replacements planned. Academic radiochemistry facilities lack the capacity for timely manufacture of all the radiopharmaceuticals that might be useful. Even the commercial supply of recognized products such as [^131^I]MIBG has been erratic and unreliable for clinical users, with very late cancellation of orders to the detriment of patient care. This was a major factor in the premature closure of the VERITAS clinical trial previously mentioned. Despite the proven clinical value of [^131^I]MIBG, the only supplier recognized by the U.K. Medicines and Healthcare Products Regulatory Agency, GE HealthCare, stopped supplying it across Europe at the end of 2024. One commercial supplier remains in Europe; whether there is sufficient production capacity for all users remains unclear. Previously, there were 2 [^131^I]MIBG suppliers in North America. Production of a no-carrier-added formulation has been discontinued by Lantheus, and therefore only 1 supplier remains for all of North America. Additional sources of production would ensure an ongoing, reliable supply for this critical medication across both continents.

A multistakeholder group, Radionuclides for Health UK, has been formed to raise awareness of these difficulties and to campaign for resources for better radionuclide and radiopharmaceutical provision. Its publication *Radionuclide Supply in the UK: A Path to a Cancer Breakthrough* sets out a strategy to address this issue (53).

### Patient and Public Involvement and Engagement

Children with neuroblastoma are the focus of our efforts to improve treatment, and so it is crucial that we listen to the views of their parents who advocate for them (54). Charities are central to this and work with clinical trial groups both in the U.K. and internationally. Patient advocates should be included in developing research priorities and in the design and delivery of clinical trials. In addition, advocates can play an important role in expanding access to theranostics facilities such that more patients can be treated closer to home. As new facilities are developed, advocates can lend the patient’s voice to the design of patient and family rooms to maximize comfort during therapy.

### Clinical Service Delivery

Hospitals that provide theranostics for children with neuroblastoma must be appropriately equipped and staffed, not simply to provide excellent technical imaging and therapy but also to provide holistic family-centered care. Many families travel long distances and are away from home for several weeks at a time. Just as every child is different, so families are different, with varying levels of support available. Adults are required to act as comforters and carers, and their personal radiation exposure must be kept as low as reasonably achievable. They, as well as ward staff, need specific radiation protection guidance and monitoring. Specialist staff, such as therapeutic radiographers, are essential to deliver high-quality service.

## DISCUSSION

**FIGURE 2.**
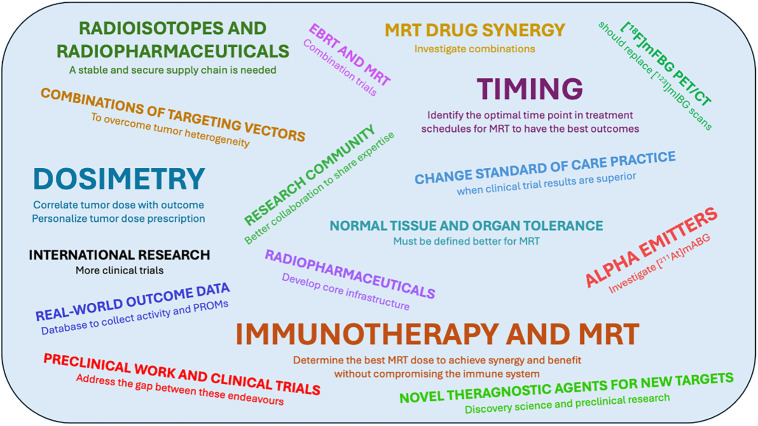
Word cloud showing ideas about where future efforts should be focused. Larger font indicates greater number of responses. EBRT = external-beam radiotherapy; mABG = metaastatobenzylguanidine; mFBG = metafluorobenzylguanidine; PROMs = patient-reported outcome measures.

This symposium brought together an international group of over 40 individuals from a wide range of professional backgrounds, and also patient advocates, all interested in further developing theranostics for neuroblastoma. There were active discussions, and new preliminary research ideas were generated, which will be considered further. Figure 2 illustrates the range of priorities that participants identified.

Neuroblastoma is genetically and phenotypically diverse. This disease heterogeneity is important, and so individualization should be considered when selecting the most appropriate therapy. One strength of molecular imaging is to identify the better target for the individual patient. The main conclusions were that efforts are needed both to address logistic constraints and to promote further research in imaging and treatment to optimize clinical outcomes. Clearly, increased funding is important, as both aspects require significant investment.

Radiopharmaceutical production needs strengthening, especially for orphan drugs that may be of great value for a small number of patients but are not commercially profitable. Similarly, a greater academic radiopharmacy capacity is needed to prepare novel compounds in a timely way for research. Additional clinical facilities for treating young children would be advantageous, as currently there are too few, resulting in geographic inequity of service provision.

Research priorities include both preclinical endeavors to evaluate innovative ideas and an expanding portfolio of clinical trials to assess different strategies to improve results including radiosensitization, radiation and immunotherapy combinations, and novel agents. These will establish evidence and guide sequencing of therapies.

Given the multidisciplinary effort required to move the field forward and to implement theragnostic advances, it is essential that leading individuals from different specialties work together with patient advocates to raise awareness of the potential of MRT and lobby nationally and through international collaboration for better resourcing.

## CONCLUSION

Theranostics is an important area of research and clinical practice as part of the multimodality treatment of neuroblastoma. International multidisciplinary collaboration is the key to advancing understanding of the use of radiopharmaceuticals in the diagnosis and treatment of this childhood cancer of unmet need.

## DISCLOSURE

The Children’s Cancer Research Fund (U.K. Charity no. 1046278) sponsored the workshop, its scientific advisory board planned the content, and AstraZeneca, Joseph’s Smile, Recordati Rare Diseases, and Siemens Healthineers provided generous financial support. Peter Gawne is supported by Neuroblastoma UK. Mark Gaze is supported by the National Institute for Health Research University College London Hospitals Biomedical Research Centre and by the Radiation Research Unit at the Cancer Research UK City of London Centre Award (C7893/A28990). Helen Bryant is supported by Children with Cancer and CCLG–Little Princess Trust. Sally George is supported by Cancer Research UK (CRUK) and CCLG–Little Princess Trust. Kyle Matchett is supported by Higher Education Authority (HEA) and CCLG–Little Princess Trust. Simon Wan is supported by the National Institute for Health Research University College London Hospitals Biomedical Research Centre. The MINT clinical trial is supported by Solving Kids Cancer UK, the SickKids Foundation, and the Penelope Neuroblastoma Foundation. Peter Gawne reports travel expenses from Recordati Rare Diseases. Steven DuBois reports consulting fees from Amgen, Bayer, InhibRx, and Jazz and travel expenses from Loxo, Roche, and Salarius. No other potential conflict of interest relevant to this article was reported.

## Notes

KEY POINTS **QUESTION:** How can research and clinical practice in nuclear medicine imaging and MRT for neuroblastoma be enhanced and improved for patient benefit? **PERTINENT FINDINGS:** Carefully focused multiprofessional and multidisciplinary collaboration may determine clearer priorities for research and clinical practice in neuroblastoma theranostics. **IMPLICATIONS FOR PATIENT CARE:** Use of novel radiopharmaceuticals in clinical trials, aligned with the use of innovative technologies, may improve outcomes in this pediatric cancer of unmet need.
